# Evaluation of Programmed Death - Ligand 1 (Pd-L1) Expression in Head and Neck Squamous Cell Carcinoma

**DOI:** 10.30699/ijp.2023.1988955.3057

**Published:** 2023-06-20

**Authors:** Aarya Unnikrishnan, Vijaya Basavaraj

**Affiliations:** *Department of Pathology, JSS Medical College, JSS Academy of Higher Education and Research, Mysuru, Karnataka, India*

**Keywords:** Head and neck, Programmed death ligand-1, Squamous cell carcinoma, Tumor-infiltrating lymphocytes

## Abstract

**Background & Objective:**

Head and Neck Squamous Cell Carcinoma (HNSCC) is a highly frequent malignancy worldwide and is also the leading cause of death. The prognosis for individuals with HNSCC remains dismal, with a five-year survival rate of less than 50%. The novel anti-PD-L1 immunotherapy is found to be promising, and immunohistochemistry (IHC) has been established as a reliable method for patient stratification. We intend to evaluate the prognostic significance of the expression of programmed death ligand-1 (PD-L1) in HNSCC and determine its association with clinicopathological variables.

**Methods:**

A total of 50 cases of biopsy-confirmed HNSCC were studied in a tertiary hospital between Dec 2020 and June 2022. The specimens were tested for PD-L1 IHC expression with antibody clone CAL-10 (Biocare) and scored by Combined Positive Score (CPS). The association between PD-L1 expression and clinicopathological variables was evaluated.

**Results:**

PD-L1 was positive in 92% of the cases, and a significant association (*P*= 0.024) was seen between PD-L1 expression and tumor-infiltrating lymphocytes (TILs). PD-L1 did not show any significant association with patient demographics, tumor site, grade, or stage.

**Conclusion:**

In the present study, evaluation of the immunohistochemical expression of PD-L1 on the tumor cells and TILs in HNSCC revealed a high prevalence of PD-L1 expression. PD-L1 IHC studies for patient selection for immunotherapy would be a promising technique. Frequent PD-L1 expression in tumors with significant TILs may be useful in identifying patients who may benefit from anti-PD-1/PD-L1 therapy.

## Introduction

Head and neck cancers (HNCs) are the leading cause of cancer mortality, especially among men (1). The most common pathological type of HNC is squamous cell carcinoma (2). The pathogenesis of HNSCC is multifactorial and includes human papillomavirus (HPV) infection, smoking, areca nut chewing, alcohol abuse, and an extended life expectancy (2-4). Despite recent advances in treatment modalities, the rise in morbidity and mortality continues to increase worldwide due to late diagnosis. Despite having benefits, combination therapy has shown serious complications and life-altering toxicity (3). HNSCC has a poor prognosis, with a five-year survival rate of approximately fifty percent (2). Recurrence of the tumor and metastasis within a period of 3 years has been seen in more than 50% of HNSCC patients (2). Cancer immunotherapy has become one of the most significant therapies, and anti-PD-L1 immunotherapy is a promising therapy that we look forward to (2, 3, 5).

 PD-1/CD279 is a T cell co-inhibitory receptor belonging to the CD28 family that expresses on various immune cells, such as tumor-infiltrating CD8+ T cells, CD4+ T cells, activated monocytes, B lymphocytes, natural killer cells, and dendritic cells. It acts as a negative regulator of the immune system, functions by forming a complex with its ligands, and helps in maintaining immune homeostasis (2).

 PD-L1 (B7-H1) and PD-L2 are the two known ligands of PD-1. PD-L1 is expressed in multiple types of cells, which include T cells, B cells, antigen-presenting cells (APCs), monocytes, macrophages, non-immune cells such as endothelial cells, keratinocytes, corneal cells, and various tumor cells (2, 3, 5).

 The PD-L1/PD-1 mechanism has been used by tumor cells to suppress immune surveillance and facilitate tumor progression (6). Along with PD-1, PD-L1 also inhibits T cells by binding to CD80 (2, 7). PD-L1 expression on the tumor cells, the presence of intratumoral infiltrating immune cells (I-TIICs), and PD-1 receptor expression in TILs have crucial associations (8). Therefore, the patients with I-TIICs expressing PD-L1 may particularly benefit from the use of immune checkpoint blockade (6).

 Many studies have been carried out about the expression of PD-1 and PD-L1 in tumors and their association with the clinicopathological parameters of HNSCC (2). The clinical response to PD-L1-directed immunotherapy was found to have a close association with PD-L1 expression. So far, apart from the quantification of PD-L1 with IHC, there aren’t any predictors that may help in the stratification of the patients who could benefit from checkpoint inhibitors (9).

 We intend to evaluate the prognostic significance of the expression of PD-L1 in HNSCC and determine its association with clinicopathological variables. Our study is an attempt to highlight the near-future possibility of the role of PD-L1 as a predictive biomarker for immunotherapy response in patients with HNSCC.

## Material and Methods

The present study was undertaken in the Department of Pathology in a tertiary care center in India. A total of 50 cases of biopsy-confirmed HNSCC was studied, which included both prospective and retrospective cases over a period of 5 years between 2018 and 2022.

Inclusion criteria: All cases of HNSCC who underwent primary surgery, including neck dissection, were included in the study.

Exclusion criteria: Small biopsies of suspected HNSCC sent for diagnostic confirmation were excluded from the study.

 In retrospective cases, all of the clinical data was recovered, and the sections were extracted from the department's paraffin blocks. The clinical history was obtained, and the tissue was routinely processed in prospective cases. The sections prepared were subsequently subjected to hematoxylin and eosin (H&E) staining and PD-L1 staining. Routine H&E stained sections were analyzed for pathological variables. The tumor was histologically graded according to Broder's classification. Tumor, nodal status, and metastasis (TNM) classification were used for pathological staging. HNSCC tissue specimens that were tested for PD-L1 expression were scored using the Combined Positive Score (CPS), and relevant cutoff scores taken were CPS <1, CPS ≥1, and CPS ≥20. The study was carried out after receiving approval from the institutional ethics committee.

**IHC Staining Procedure for PD-L1:**

The formalin-fixed sections on the positively charged slide were kept in the hot air oven at 60°C for 20 minutes. Before staining, deparaffinization was done using xylene, and rehydration of the 3-µm slides was done using graded alcohols. Antigen Retrieval was done by placing the slides in the Antigen Retrieval Solution ((Biocare Medical, USA made; Decloaking Chamber, and EDTA Solution pH9) in the Decloaking chamber, and the program was set at 110°C for 20 min. After antigen retrieval, the slides were cooled in distilled water and kept in the buffer solution for 5 minutes.

 The peroxide block was applied for 5 minutes, followed by the sniper protein block for 10 minutes. Incubation of the sections was done with the primary antibody CAL10, PD-L1 (M/s Biocare Medicals USA) for 60 minutes. Bound antibodies were further incubated with MACH-1 HRP polymer for 30 minutes. Subsequently, visualization of the antibody was achieved following the addition of betazoid DAB chromogen for 5 minutes. CAT hematoxylin counterstain was applied for 1 minute. Tonsil tissue was used as the positive control for staining.

 PD-L1 expression in HNSCC was determined by using CPS. It is the number of PD-L1 staining cells (tumor cells, lymphocytes, and macrophages) divided by the total number of viable tumor cells, multiplied by 100. PD-L1 expressions of <1, ≥1, and ≥20 were recorded. Two observers assessed the PD-L1 expression with CPS evaluation in the stained slides of HNSCC in a blinded fashion without knowledge of clinical data. CPS <1 is regarded as negative, whereas CPS ≥1 and ≥ 20 are regarded as positive (10).

**Data Analysis:**

 The statistical analysis (Pearson Chi-Square test) to analyze the correlation between PD-L1 expression and clinicopathological parameters was performed using the Statistical Package for Social Sciences (SPSS) computer program version 29. A P-value<0.05 was considered statistically significant. Interobserver variability between the two observers was calculated using Cohen’s kappa coefficient. The percentage was used for categorical data, and graphs were generated using WPS Office for UWP. 

## Results

TMajority of the patients (13/50) were in the age range of 50-59 years, with a mean age of 58.98 years, and the patients’ ages ranged from 32 to 88 years. The M:F ratio was 3.17:1, with a higher prevalence in males. Thirty-one cases (62%) reported a history of tobacco abuse, while the remaining nineteen (38%) cases were non-smokers. Twenty-seven of the 31 cases with a tobacco history were male, while four were female. Thirty-one cases (62%) had a tumor in the oral cavity, 17 (34%) in the larynx, and 2 (4%) in the hypopharynx. Nine cases (18%) were well differentiated, 36 (72%) were moderately differentiated, and five (10%) cases were poorly differentiated SCC. The image depicting moderately differentiated HNSCC is shown in Figure 1. Lymphovascular invasion (LVI) was found in 28 (56%), and perineural invasion (PNI) in 11 (22%). The image depicting PNI is shown in Figure 2.

 The distribution of mononuclear inflammatory cells (MICs) was classified into minimal/mild and moderate/dense. Thirty-six cases (72%) had moderate-to-dense TILs or MICs. The image depicting dense TILs is shown in Figure 3. The tumor stage (T) of pTNM is determined based on tumor size. Twenty-one cases (42%) were in tumor stage 3, 15 cases (30%) were in stage 2, 8 cases (16%) were in stage 4, and 6 cases (12%) were in stage 1. At the time of presentation, 25 cases (50%) had metastases to the cervical lymph nodes, while the remaining 25 cases (50%) had no metastases. Out of the twenty-five cases positive for lymph nodes, five (20%) belonged to the N1 stage, 11 (44%) belonged to the N2 stage, and nine (36%) were in the N3 stage. Sixteen (64%) of the 25 metastatic lymph nodes showed extranodal extension. Most of the cases were presented at stage IV (24/50 cases, 48%), followed by stage III (12/50 cases, 24%), stage II (9/50 cases, 18%), and stage I (5/50 cases, 10%).

Forty-six (92%) of the 50 cases showed positivity for the PD-L1 IHC marker. Four cases were negative for PD-L1 expression. A CPS in the range ≥1 to <20 was seen in 23 cases, and ≥20 was observed in another 23 cases, accounting for 46% each. Immunohistochemical staining images showing the PD-L1 expression of tumor cells and TILs are shown in Figure 4.

 The association of PD-L1 expression with clinicopathological variables in the 50 HNSCC patients is as shown in Table 1. In the CPS category of ≥20, 74% of the cases belonged to tumors of the oral cavity, which was statistically significant with a P-value of 0.001. Moderately differentiated tumors with CPS ≥1 and CPS ≥20 accounted for 86.9% and 65.2%, respectively, and showed a statistical correlation with CPS ≥1 and CPS ≥20 with a P-value of 0.001 and 0.005, respectively. Seventy-eight percent of the cases were negative for PNI and PNI negatively correlated with PD-L1 expression.

Of the 36 cases (72%) having moderate to dense tumor-infiltrating lymphocytes, 15 (41.7%) had CPS ≥20, and 20 (55.6%) had CPS in the range ≥1 to <20. One case (2.8%) was PD-L1 negative. In the remaining 14 cases with minimal to absent tumor-infiltrating lymphocytes, 8 (57.1%) had CPS ≥20, 3 (21.4%) were in the range of CPS ≥1 to <20, and 3 (21.4%) were negative with CPS <1. The P-value, 0.024 showed statistical significance. Of the cases with CPS in the range ≥1, 87% cases had moderate to dense TILs while 13% had minimal to mild TILs. The P-value (0.001) showed a significant association between moderate-to-dense TILs and CPS ≥1.

**Interobserver Variability:**

 An assessment of interobserver variability between the two observers was carried out. The scoring discrepancy was found only in 1 case. PD-L1 expression was found to have an almost perfect interobserver agreement as assessed by Cohen's kappa coefficient, which had a value of 0.965.

**Table 1:** Association between PD-L1 expression and the clinicopathological variables in HNSCC

Clinicopathological variables		Number of cases	CPS			P-value
			<1	≥1 to <20	≥20	
Age	< 60	26(100%)	1(3.8%)	13(50%)	12(46.2%)	0.507
	≥ 60	24(100%)	3 (12.5%)	10(41.7%)	11(45.8%)	
Gender	Male	38(100%)	3(7.9%)	20(52.6%)	15(39.5%)	0.225
	Female	12(100%)	1(8.3%)	3(25%)	8(66.7%)	
Tobacco Usage	Present	31(100%)	2(6.5%)	16(51.6%)	13(41.9%)	0.578
	Absent	19(100%)	2(10.5%)	7(36.8%)	10(52.6%)	
Tumor site	Larynx	17(100%)	2(11.8%)	11(64.7%)	4(23.5%)	0.148
	Oral cavity	31(100%)	2(6.5%)	12(38.7%)	17(54.8%)	
	Hypopharynx	2(100%)	0(0.0%)	0(0.0%)	2(100%)	
Tumor grade	Well differentiated	9(100%)	2(22.2%)	2(22.2%)	5(55.6%)	0.112
	Moderately differentiated	36(100%)	1(2.8%)	20(55.5%)	15(41.7%)	
	Poorly differentiated	5(100%)	1(20.0%)	1(20.0%)	3(60.0%)	
LVI	Absent	22(100%)	2(9.1%)	8(36.4%)	12(54.5%)	0.478
	Present	28(100%)	2(7.1%)	15(53.6%)	11(39.3%)	
PNI	Absent	39(100%)	4(10.3%)	17(43.6%)	18(46.2%)	0.508
	Present	11(100%)	0(0.0%)	6(54.5%)	5(45.5%)	
T Stage	T1	6(100%)	0(0.0%)	2(33.3%)	4(66.7%)	0.142
	T2	15(100%)	0 (0.0%)	6(40%)	9(60%)	
	T3	21(100%)	4(19%)	9(42.9%)	8(38.1%)	
	T4	8(100%)	0 (0.0%)	6(75%)	2(25%)	
N Stage	N0	25(100%)	2(8%)	11(44%)	12(48%)	0.289
	N1	5(100%)	0 (0.0%)	1(20%)	4(80%)	
	N2	11(100%)	2(18.2%)	7(63.6%)	2(18.2%)	
	N3	9(100%)	0 (0.0%)	4(44.4%)	5(55.6%)	
ENE	Absent	9(36%)	0(0.0%)	3(25%)	6(54.5%)	0.183
	Present	16(64%)	2(100%)	9(75%)	5(45.5%)	
TILs	Minimal/Mild	14(100%)	3(21.4%)	3(21.4%)	8(57.1%)	0.024* Significant
	Moderate/Dense	36(100%)	1(2.8%)	20(55.6%)	15(41.7%)	
Disease Stage	I	5(100%)	0(0.0%)	2(40%)	3(60%)	0.453
	II	9(100%)	0(0.0%)	3(33.3%)	6(66.7%)	
	III	12(100%)	2(16.7%)	4(33.3%)	6(50%)	
	IV	24(100%)	2(8.3%)	14(58.3%)	8(33.3%)	

*P-value < 0.05 between the three categories for a given variable. LVI - Lymphovascular invasion, PNI - Perineural invasion, T stage - Tumor stage, N stage - Nodal stage, ENE - Extranodal extension, TILs - Tumor infiltrating lymphocytes, CPS - Combined positive score.

## Discussion

HNSCC, especially cancers of the lip and oral cavity, is the major cause of cancer mortality, especially among Melanesian and South Central Asian men (1). HNSCC has a poor prognosis, with a five-year survival rate of forty to fifty percent (2). Approximately 10% of HNSCC patients would have developed metastasis at the time of presentation (1).

 In recent years, tumor immunotherapy has slowly emerged as the most promising anti-tumor treatment (5). The introduction of anti-PD-1 immunotherapy in the treatment of HNSCC has marked a turning point (11). Studies have shown that expression of PD-L1 on tumor cells may have prognostic significance, and hence pembrolizumab was approved in 2019 as 1st line therapy in HNSCC patients with a CPS of ≥1 (4, 12). Certain studies have shown that PD-L1 positivity in HNSCC correlates with the potency of the drugs (5). The promising anti-PD-1 and/or PD-L1 therapy is efficacious and produces less toxicity in advanced HNSCC patients (2).

 Evaluation of the PD-L1 expression can provide a better understanding of the biological behavior of head and neck SCC (2). Hence, PD-L1 signaling represents a valuable therapeutic target for HNSCC immunotherapy. So far, apart from the quantification of PD-L1 with IHC, there aren’t any predictors that can be used to pinpoint cancer patients with higher expression of the PD-L1 marker and who could benefit from checkpoint inhibitors (9).

 In the current study, the mean age of the presentation of the cases was 58.98 years. Most of the cases were in the sixth decade and showed concordance with other studies (13–17). The association of PD-L1 with age showed no statistical significance. However, Ngamphaiboon N* et al.* observed a significant association between PD-L1 expression and older age (*P*<0.001) (18). Men outnumbered women with an M:F ratio of 3.17:1, which was concordant with the literature. The gender difference was not significantly associated with PD-L1 expression and was in agreement with other studies in the literature (13, 19). Satgunasheelan* et al.* observed higher PD-L1 expression in females than males (20).

 The most significant and well-established cause of HNCs is tobacco exposure, according to Hashim D* et al.* (21). In the present study, tobacco abuse was noted in 62% of the cases. The history of tobacco in various studies ranged from about 56% to 85%. No statistical association with PD-L1 expression was noted, and the same was observed in other studies in the literature (14, 22, 23).

 The most common site of occurrence of HNSCCs varies depending upon the geographic area of disease occurrence. Oral cavity carcinomas are more common in Melanesia and South Central Asia (1). In the current study, the majority of the tumors were found in the oral cavity (62%), which was seen in other studies as well (5, 15, 23). A few other studies, like those of Schneider S* et al.*, Kim HS* et al.*, and Wusiman D* et al.*, showed tumors predominantly in the oropharynx, tonsil, and larynx, respectively (5, 13–15, 17, 24).

 In the CPS category of ≥20, 74% of the cases belonged to tumors of the oral cavity, which was statistically significant (*P*= 0.001). Although the tumor sites, i.e., the oral cavity, larynx, or hypopharynx, did not show statistical significance in the expression of the PD-L1 marker, a statistically significant association was seen in positive CPS ≥20 with oral cavity tumors showing a high incidence. A significant association was found between PD-L1 expression and tumor site by Wusiman* et al.* (17).

 Based on the tumor grade, 72% of the cases were moderately differentiated, 18% were well-differentiated, and 10% of the cases were poorly differentiated. A significant statistical association with histological grade was not observed in the present study and is consistent with other studies in the literature (5, 15, 18). However, of the PD-L1 positive cases that showed CPS in the range ≥1 to <20, 86.9% were moderately differentiated, and of the PD-L1 positive cases that showed CPS ≥20, 65.2% were moderately differentiated. Hence, a statistically significant association was achieved between positive CPS scores of ≥1 and ≥20 and moderately differentiated tumors. At the CPS cutoffs of ≥1 and ≥20, the P-values were 0.001 and 0.005, respectively. 

 In the present study, the majority of the cases presented at tumor stage 3 (42%), followed by stage 2 (30%). The T stage did not show a significant association with PD-L1 expression and was in concordance with other studies seen in the literature. However, Wusiman D* et al.* found an association between PD-L1 expression and tumor stage with a P-value of 0.022 (17). We classified the nodal stage as node-negative cases (N0) and node-positive cases (N+). Node-negative cases were 25, and N+ cases were 25. Of the cases with positive PD-L1 staining, 23 cases were N0 and 23 cases were N+. Twenty-three cases each of node-negative and node-positive showed PD-L1 expression, and no pathologically significant association between the nodal status and PD-L1 expression was seen, which was consistent with the study by Mishra PS* et al.* (15). Schneider S* et al.* noted a significant association between PD-L1 expression and N staging, where N staging was classified as N0 and N+. A statistically significant correlation was seen in the pattern of expression of the primary tumor and corresponding lymph nodes (13). In the present study, most of the cases belonged to stage IV, which was in agreement with studies by Schneider S* et al.* and Kim HS* et al.* (13, 14). No association was found between PD-L1 and the disease stage.

 The distribution of tumor-associated MICs/TILs was classified into minimal/mild and moderate/dense. Seventy-two percent of cases had moderate-to-dense TILs or MICs. Statistical significance (*P*=0.024) was achieved between PD-L1 and TILs and was consistent with the study by Lenouvel* et al.* (p value = 0.029) (20). According to the study by Satgunasheelan* et al.*, expression of PD-L1 was considerably higher in cancers having an inflammatory nature, with a p value of 0.001 (19). In the present study, of the cases with CPS in the range ≥1 to <20, 87% had moderate to dense TILs, while 13% had minimal to mild TILs. The p value (0.001) showed a significant association in the CPS range ≥1 to <20 with TILs, with moderate-to-dense TILs showing a higher incidence. The immune response created in the tumor microenvironment as a result of immune evasion by the tumor cells has been shown to play a major role in cancer immunotherapy (25). Therefore, along with PD-L1 IHC expression, TILs analysis may provide a helpful additional parameter for assessing tumor immune response, though it needs further prospective investigations (26).

 LVI was found in 56% and PNI was found in 22% of cases. LVI and PNI did not show any statistical significance and were concordant with the study by Satgunasheelan* et al.* (18). Seventy-eight percent of the cases were negative for PNI, and PNI negatively correlated with PD-L1 expression.

 The PD-L1 expression in different studies varied from 36% to 92.5%, as shown in Table 2. In the current study, PD-L1 expression was seen in 92% of the cases. A CPS ≥1 was present in 46 (92%) cases, and a CPS of ≥20 was present in 23 (46%). Twenty-three (46%) cases were present in the CPS range ≥1 to <20. The high PD-L1 expression is in agreement with studies by Wusiman D* et al.* and Guerini Rocco* et al.* (11, 17).

**Table 2:** Comparison of PD-L1 Expression in Various Studies

Author	Total No. of Cases	Cut-off score	PD-L1 Expression	Clone	Tumor site
Schneider S* et al.*^13^	125	≥5	36%	5H1	HNSCC
Kim HS* et al.*^14^	133	≥20	68%	5H1	OSCC
Wusiman D* et al.*^17^	119	≥1 ≥20	89.9% 43.7%	22C3	HNSCC
Ngamphaiboon N* et al.*^18^	203	≥5	80%	SP142	HNSCC
Mishra PS* et al.*^15^	93	≥1 ≥50	15(16.1%) 44(47.3%)	22C3	HNSCC
Chen SW* et al.*^5^	95	-	64%	Proteintech USA	HNSCC
Cho YA* et al.*^27^	45	-	87%	ab82059 Abcam UK	HNSCC
Guerini Rocco* et al.*^11^	40	≥1 ≥20	92.5% 45%	22C3 Omnis	HNSCC
		≥1 ≥20	92.5% 16%	SP263	
		≥1 ≥20	92.5% 60%	22C3 Auto L48	
Cerebelli B* et al.*^28^	43	≥1	88.4%	22C3 SP263	HNSCC
Lenouvel D* et al.*^20^	55	≥5	58%	22C3	OPSCC
De Ruiter EJ* et al.*^29^	147	≥1	45.6%	22C3 pharmDx	HNSCC
			87.1%	SP263	
			63.9%	22C3 LDT	
Present study	50	≥1 ≥20	92% 46%	CAL-10 Biocare	HNSCC

HNSCC - Head and neck squamous cell carcinoma, OSCC - Oral squamous cell carcinoma, OPSCC - Oropharyngeal squamous cell carcinoma

 The antibody clones and scoring systems have varied in different studies. PD-L1 immunohistochemistry performed with clone 22C3 using the Dako Link 48 automated platform is the only companion diagnostic indicated for the selection of cases for anti-PD-1 therapy in head and neck SCC (9, 15). The PD-L1 IHC clones, evaluation method, and cut-off scores used largely impact the PD-L1 expression levels (18). In the present study, the following clone of antibody was used: CAL10, Biocare, Source: Rabbit monoclonal; Primary antibody (M/s Biocare Medicals, USA); Secondary detection kit MACH 1, (Biocare Medicals, USA).

 In the KEYNOTE-048 study (n = 601), PD-L1 positivity was assessed using PD-L1 IHC 22C3 pharmDx for the subcategory assigned to KEYTRUDA as a single agent or to cetuximab in conjunction with chemotherapy. Eighty-five percent of the cases showed PD-L1 expression with a CPS greater than 1. Forty-three percent of the cases showed PD-L1 with a CPS under 20, which was consistent with the present study (10).

 Guerini Rocco* et al.* and de Ruiters EJ* et al.* analyzed IHC using three clones of PD-L1 antibodies (clones 22C3 Omnis, SP263, and 22C3 Auto L48 in 40 and 22C3 pharmaDx, SP263, and 22C3 LDT in 147 samples, respectively) and qualitatively compared them at the ≥1 and ≥20 cutoffs. When a cut-off of ≥1 was taken, 92.5% of the cases had shown PD-L1 positivity using both SP263 and 22C3 pharmaDx assays, which was in agreement with our study (11, 29).

 Immune checkpoint inhibitors (ICI) have shown promising results when used to treat advanced HNSCC. Pembrolizumab has been approved as first-line therapy in metastatic disease with patients having PD-L1 expression level CPS ≥ 1. In cases where the CPS ≥20, the use of Keytruda as a single agent is not recommended. Nivolumab has been approved by the FDA as a 2nd-line treatment in platinum-refractory disease (10).

 Different studies have shown highly variable PD-L1 expression in HNSCC, which might be attributable to intratumoral heterogeneity along with temporal changes (30). The PD-L1 IHC antibody clone used, the scoring system, and the cutoff score for positivity also partly contribute to the same (18). Hence, a proper understanding of the scoring system and application of clinically relevant cutoffs for identifying the patients who are eligible for anti-PD-1 therapy is essential (31).

## Conclusion

The introduction of PD-L1/PD-1 checkpoint inhibitors has been a turning point in cancer immunotherapy. Given the development of numerous approved immune checkpoint inhibitors, we attempted to evaluate PD-L1 immunohistochemical expression in HNSCC with a different clone, not so widely used, and determine its association with clinicopathological parameters.

In the present study, evaluation of immunohistochemical expression of PD-L1 on tumor cells and tumor-infiltrating immune cells in HNSCC revealed a high prevalence of PD-L1 expression (92%), using CPS, proving that PD-L1 IHC done given patient selection for immunotherapy is a promising technique. 

A statistically significant association with a p-value of 0.024 was observed between the PD-L1 score and tumor-infiltrating immune cells, suggesting that frequent PD-L1 expression in tumors with significant TILs may be useful in identifying patients who may benefit from anti-PD-1/PD-L1 therapy. Further research on a larger cohort is required to confirm our findings, as the scope and nature of the application of PD-L1 immunohistochemistry are constantly expanding.

The small sample size was a limitation of the present study. Complete follow-up data were not available, and hence the recurrence and survival analyses were not addressed.

## Funding

 This research received no specific grant from any funding agency in the public, commercial, or not-for-profit sectors.

## Conflict of Interest

There are no conflicting interests.
